# Notes from Private Practice

**Published:** 1877-09

**Authors:** 


### NOTES FROM PRIVATE PRACTICE.

*The poisonous effects of arnica.*

Mr. L——, a healthy gentleman, of excellent habits, and with no specific cachexia, slightly injured his knee by falling on the sidewalk, while attempting to reach the street cars. Partly to quiet the pain, and partly to follow a very fashionable and orthodox custom prevalent among a majority of the people, he purchased at one of our best drug stores, a two-ounce bottle of tincture of arnica, the contents of which he applied to the knee. In this application no particular attention was given to the quantity used, and some of the tincture trickled down the leg, and a small amount of it came in contact with the fingers, especially between them. During the night the affected limb, swathed in a cloth saturated with the arnica, came in contact with the inner part of the other leg, and at some time, probably while applying the lotion in the evening, the patient's fingers came in contact with his eyelids. In the morning every part of the integument which had been touched with the arnica, was slightly red and swollen—erythematous. I was immediately summoned, October 17th, and ordered salines, a cooling lotion and rest. 18th, swelling and redness increased—some fever. 19th, vesicles between fingers and upon left knee. 20th, vesicles upon portions of all the integuments (except eyelids) with which the arnica had come in contact—eczema. 22d, eye nearly well; integuments less swollen; considerable itching and burning; pustules around the margins of affected parts. 23d, intense itching and burning of the surface of the thorax and abdomen. An examination showed a profuse redness or vascular congestion of the entire surface, and the presence of what Wilson would probably call eczema erythematosum. 24th to 28th, eruption on body disappearing; crusts forming on leg; fingers nearly well. 29th to 31st, crusts clearing; pruritus disappearing; new skin looking healthy; patient discharged.

This gentleman, without doubt, from the external use of the arnica, had an acute eczema running through the different stages of hyperæmia, increased sensibility, infiltration, vesiculation, incrustation and degeneration, terminating at about the usual time. The constitutional disturbances were greater than is usual, as was shown by the eczema erythematosum, which took place the sixth day, and by the excoriated and ulcerated patches which continued for two or three weeks after his discharge. I should mention, perhaps, that some two or three years ago, I treated the same gentleman for a slight eczema on the hand, and it is perhaps in these people who have, as it were, an eczematous diathesis that this unusual susceptibility of the skin is present. Until this case occurred, in my own practice, I was ignorant of the fact that arnica applied externally ever produced poisoned effects. I considered it a domestic remedy used very generally without the advice of a physician, and supposed it perfectly harmless. I find, however, that Fox, in speaking of medicinal rashes, says, "Arnica may produce erythema and swelling of the part to which it is applied, or it may excite a real eczema." And Hebra, in his article on erythema nodosum, speaks as follows: "Some medical men, however, suppose that the tincture of arnica is a perfectly harmless remedy in erythema nodosum, and in similar affections. But I would give a friendly warning to those who advocate its use, unless, indeed, they propose to use it homœopathically, and in infinitessimal doses. In the proportion of a drop of the tincture to a pail of water, this substance may certainly be applied without any risk of doing harm. But I have in practice had abundant reason to observe that the tincture of arnica, even when much diluted, acts most injuriously upon the skin of some persons. I have frequently seen eczema or dermatitis excited by the assiduous application of lotions containing this drug in the treatment of slight bruises or sprains." In the *Medical and Surgical Journal*, of Boston, Jan. 21st, 1875, Dr. Jas. C. White reports three cases, somewhat similar to mine. His patients had received injuries of some severity, and following the ancient and honorable custom, applied arnica, which in every case produced hyperæmia, vesicles, etc., in regular order. In view of the universal belief of the harmlessness of this drug, it seems best to place on record well attested facts in regard to its poisonous effects on, at least, a certain number of those who use it.

CHAS. W. EARLE, M. D.

No. 37 Park Avenue.

---

*Atresia Vaginæ with Retention of the Menses.*

Mrs. H. came under my observation thirteen months after the birth of her first child. The attending physician in her confinement found the hymen unruptured, and he could only, with great difficulty, force the index finger into the vagina.

The labor was natural, and in its progress the hymen was ruptured without any interference.

The patient made a good recovery; no sloughing or other bad symptom followed, and the lochia, which was not very abundant, ceased in ten days. About the ninth month after delivery, she had all the symptoms of menstruation with the exception that there was no discharge.

A month later, there was again the menstrual molimen present, with pain and more general disturbance. With each succeeding month, the pain increased and was, as she described it, like colic. The pain, each time, was caused probably by the distention of the vagina, and the contraction of the uterus in its efforts to expel its contents. She suffered but little inconvenience during the intervals between the menstrual periods. Examination of the patient, four months after menstruation began, disclosed a small tumor as high as the umbilicus, which proved to be the uterus. Upon attempting vaginal examination, I found the opening occluded just within the internal labia. Inspection disclosed a firm star-like, or radiated cicatrix at the site of the hymen, or carunculæ myrtiformes. Further examination per rectum disclosed an elastic tumor, impinging upon the rectum and extending upward, beyond exploration in that direction.

There was no doubt as to the correct diagnosis of the case. The vagina had been hermetically sealed by the reunion of the forcibly ruptured hymen. The accumulated menstrual flow had distended the vagina, and pushed the uterus up out of the pelvis.

The following plan of treatment was pursued. Having introduced a catheter into the bladder, and the index finger of the left hand into the rectum as guides, a trocar and canula were then pushed through the center of the cicatrix, into the distended vagina. On the withdrawal of the trocar, there began to flow through the canula, a thick black fluid, resembling tar or treacle. The canula was then removed, and the opening dilated; first with one finger, then with the index finger of each hand, the force being applied in opposite directions. The contents were soon discharged through the ample opening with aid of a little pressure over the abdomen.

The vagina was then washed out with a weak solution of carbolic acid and warm water. The parts were kept from contracting by the introduction of a good sized glass pestle, which was retained in situ by a T bandage. The patient made a good recovery without one bad symptom. There is a question as to the safest method of discharging the retained menses; whether at once by full, free opening, or by a small one, permitting the fluid to dribble away *guttatim*. The discharge of this fluid is by no means unattended with danger, as death has followed the operation for relief of retention, caused by an imperforate hymen. The different methods of operating by free incision, or forcible dilation, to prevent contraction and re-closure, have each their advocates. In this case forcible dilation was followed by little tendency to contract.

Olina, Ill. T. J. Maxwell, M. D.

---

*A Case of Belladonna-poisoning—Recovery.*

August 3d. Was called about 10:30 A. M. to see Corda R., aged 3½ years. When I arrived the child was apparently sleeping soundly. Pulse, 130; respirations, 20 per minute; the entire surface of the body was covered with a very bright scarlatinal eruption, with occasionally livid spots of variable size. Upon lifting the eyelids, found the pupils dilated to such an extent that only a very small circle of the irides remained visible.

The mother stated that the child had a chill on the day previous, and that she had given a large teaspoonful of quinine mixture, (quinine sulph. ℨj, syrup glycyrrhiz. ℥j) at 8 o'clock, to prevent the recurrence of the chill. In a few minutes after taking the dose the child had vomited slightly, and in less than an hour after she commenced having spasms, which continued, alternated with periods of repose until my arrival, two hours and a half later.

Upon examining the supposed quinine mixture, it proved to be fluid extract of belladonna, which the mother had used locally, some time prior to this, for the purpose of suppressing a threatened mammary abscess.

The diagnosis being settled, I gave the patient a teaspoonful of ground mustard, mixed with water, repeating the dose in a few minutes, but the imperfect deglutition rendered the administration very difficult. It was followed, however, in a short time by rather imperfect emesis. I then determined to test the antidotal powers of opium.

At 11 A. M. injected $\frac{1}{8}$ grain of acet. morph. hypodermically in right arm, and gave a teaspoonful of a strong solution of tannic acid by the mouth every half hour. Patient slept soundly directly after the injection, except frequent slight subsultus, and the slightest touch on the finger tips would produce profound digital flexion. Pulse 126, during sleep; 2 P. M., patient aroused voluntarily, talks a great deal in a thick, gruff voice. Rash entirely disappeared. Right pupil slightly contracted; left very large; very restless; injected $\frac{1}{12}$ gr. acet. morph. in left arm; continued solution of tannin.

4. P. M. Very restless; wants to walk about, but is unable to stand alone, becoming delirious, laughing wildly one moment, screaming and clutching with fright the next. Muscular incoördination in reaching for anything. Will overreach or fall short of the object; has lost all idea of distance; power of articulation destroyed. Tongue appears greatly swollen.

Rash has appeared again over the entire body, more marked at flexion of joints. Pupils intensely dilated; morbid sensitiveness to sounds and objects; is terribly frightened at objects lying about. Afraid of visitors, shrinking from them and screaming wildly; insatiable thirst; motions and calls for water incessantly, but swallows it with difficulty.

6 P. M. No better; injected $\frac{1}{8}$ grain acet. morph. in right arm.

8 P. M. Fully under influence of last injection; resting, except occasional tossing and rolling about; subsultus tendinum when quiet; pulse, 130.

10 P. M. Aroused patient—still wild and grasping at everything. Pupils slightly contracted, with diverging strabismus. Pulse, 128; respirations very slow; allowed patient to sleep again.

At midnight patient sleeping soundly; pulse, 126: respirations quite slow, with crowing sound on inspiration; becomes very rigid when aroused; relaxed when sleeping; rash disappeared again, giving solution of tannin every hour; roused the patient occasionally until 2 A. M., when she awoke—Talks much; articulation more distinct; calls constantly for water; gave olei olivae, olei ricini, āā, a teaspoonful every hour; discontinued solution of tannin and injected $\frac{1}{12}$ gr. acet. morph. in left arm.

4 A. M. Roused up and passed a large quantity of dark colored urine; pulse, 115; profuse perspiration with warm surface; remained about the same until 8 A. M., when the bowels operated freely.

10 A. M. Patient roused up much improved; disposed to play; pupils almost normal, dilating readily in the dark; contracting tardidly, when exposed to light; voice nearly normal; pulse, 96.

12 M. Twenty-eight hours after taking the belladonna all traces of it have disappeared; pulse, 90; pupil normal, but dilating readily; everything appears to indicate a rapid and permanent recovery.

Oakland, Ill. J. D. Whitley, M.D.

